# *Bacillus* and *Streptomyces* spp. as hosts for production of industrially relevant enzymes

**DOI:** 10.1007/s00253-023-12900-x

**Published:** 2024-01-30

**Authors:** Sandra Vojnovic, Ivana Aleksic, Tatjana Ilic-Tomic, Milena Stevanovic, Jasmina Nikodinovic-Runic

**Affiliations:** Institute of Molecular Genetics and Genetic Engineering, University of Belgrade, Vojvode Stepe 444a, 11042 Belgrade 152, Serbia

**Keywords:** *Bacillus*, *Streptomyces*, Enzyme, Expression, Biocatalysis

## Abstract

**Abstract:**

The application of enzymes is expanding across diverse industries due to their nontoxic and biodegradable characteristics. Another advantage is their cost-effectiveness, reflected in reduced processing time, water, and energy consumption. Although Gram-positive bacteria, *Bacillus*, and *Streptomyces* spp. are successfully used for production of industrially relevant enzymes, they still lag far behind *Escherichia coli* as hosts for recombinant protein production. Generally, proteins secreted by *Bacillus* and *Streptomyces* hosts are released into the culture medium; their native conformation is preserved and easier recovery process enabled. Given the resilience of both hosts in harsh environmental conditions and their spore-forming capability, a deeper understanding and broader use of *Bacillus* and *Streptomyces* as expression hosts could significantly enhance the robustness of industrial bioprocesses. This mini-review aims to compare two expression hosts, emphasizing their specific advantages in industrial surroundings such are chemical, detergent, textile, food, animal feed, leather, and paper industries. The homologous sources, heterologous hosts, and molecular tools used for the production of recombinant proteins in these hosts are discussed. The potential to use both hosts as biocatalysts is also evaluated. Undoubtedly, *Bacillus* and *Streptomyces* spp. as production hosts possess the potential to take on a more substantial role, providing superior (bio-based) process robustness and flexibility.

**Key points:**

• *Bacillus and Streptomyces spp. as robust hosts for enzyme production.*

• *Industrially relevant enzyme groups for production in alternative hosts highlighted.*

• *Molecular biology techniques are enabling easier utilization of both hosts.*

**Supplementary Information:**

The online version contains supplementary material available at 10.1007/s00253-023-12900-x.

## Introduction

The demand for enzyme production to meet the requirements of the food, agricultural, and pharmaceutical industries is steadily increasing, particularly as we move towards a bioeconomy. Today, thanks to biotechnological development, many enzymes are produced as recombinant proteins in model organisms like bacteria, yeasts, insect, and human cell lines (Lipońska et al. 2019). Molecular tools allowed redesign and production of the desired enzymes and their variants. Advancements in DNA technology, combined with sophisticated bioprocessing techniques, have enabled the large-scale production of enzymes as purified and well-characterized preparations. This enabled the wide application of enzymes in numerous industrial products and processes, such as detergents, food and beverages, animal feed, textile and leather, pulp and paper, bioethanol, and wastewater treatment. Enzymes also play a crucial role in a range of specialized domains, including pharmaceuticals, research and biotechnology, diagnostics, and biocatalysis. As of 2022, the global enzymes market was valued at approximately $12.1 billion, and it is projected to reach $16.9 billion by the year 2027, exhibiting a compound annual growth rate (CAGR) of 6.8% (Markets and Markets 2022, Tatta et al. 2022).

Today, microorganisms serve as the primary source of enzymes, surpassing plants and animals, while the production of hydrolases (proteases and lipases) holds the dominant position, followed by various carbohydratases, primarily amylases and cellulases (Arbige et al. 2019). Out of 70 commercially available microbial enzymes (https://www.sigmaaldrich.com/; Table S1), approximately 56% are of bacterial origin, 40% of fungal, and 4% are coming from *Archaea*. Among these enzymes, only 24% are recombinantly produced exclusively in the *Escherichia coli* host (Table S1), suggesting the preference for enzyme production in native hosts in optimized growth media. Approximately one-third of the screened enzymes coming from bacteria are actually coming from *Bacillus* and *Streptomyces* spp., eight and five from each group respectively, highlighting the importance of these Gram-positive microorganisms as a source of industrially relevant enzymes. In regard to enzyme type, proteases coming from these species are the most abundant, while *Bacillus* is also a prolific producer of esterases and chitinase from *Streptomyces griseus* made it to the market (Table S1).

Aerobic bacteria, belonging to *Bacillus and Streptomyces* genera, commonly found in soils and many different ecological niches, are known for their ability to secrete multiple hydrolytic enzymes that enables them degradation of complex organic substrates and thus survival in complex environments. Over the past few decades, they have also emerged as the appropriate microbial chassis for protein production and as an alternative platform for recombinant protein production (Kim et al. 2020; Hwang et al. 2021; Yang et al. 2021). Accumulated know-how related to production of recombinant proteins is documented in numerous review papers, describing crucial factors that impact the expression in *Bacillus or Streptomyces* spp. as distinctive hosts. The purpose of this mini-review is to conduct a direct comparison between the two expression hosts, highlighting their individual strengths in particular scenarios where bio-based production processes are preferred. Also, our intention is to assist scientists who are entering into the field of recombinant enzyme production by presenting alternative microbial hosts as viable options, alongside the traditionally utilized *E. coli*. This will offer them an opportunity to explore and consider other potential hosts for their research endeavors.

**Fig. 1:**
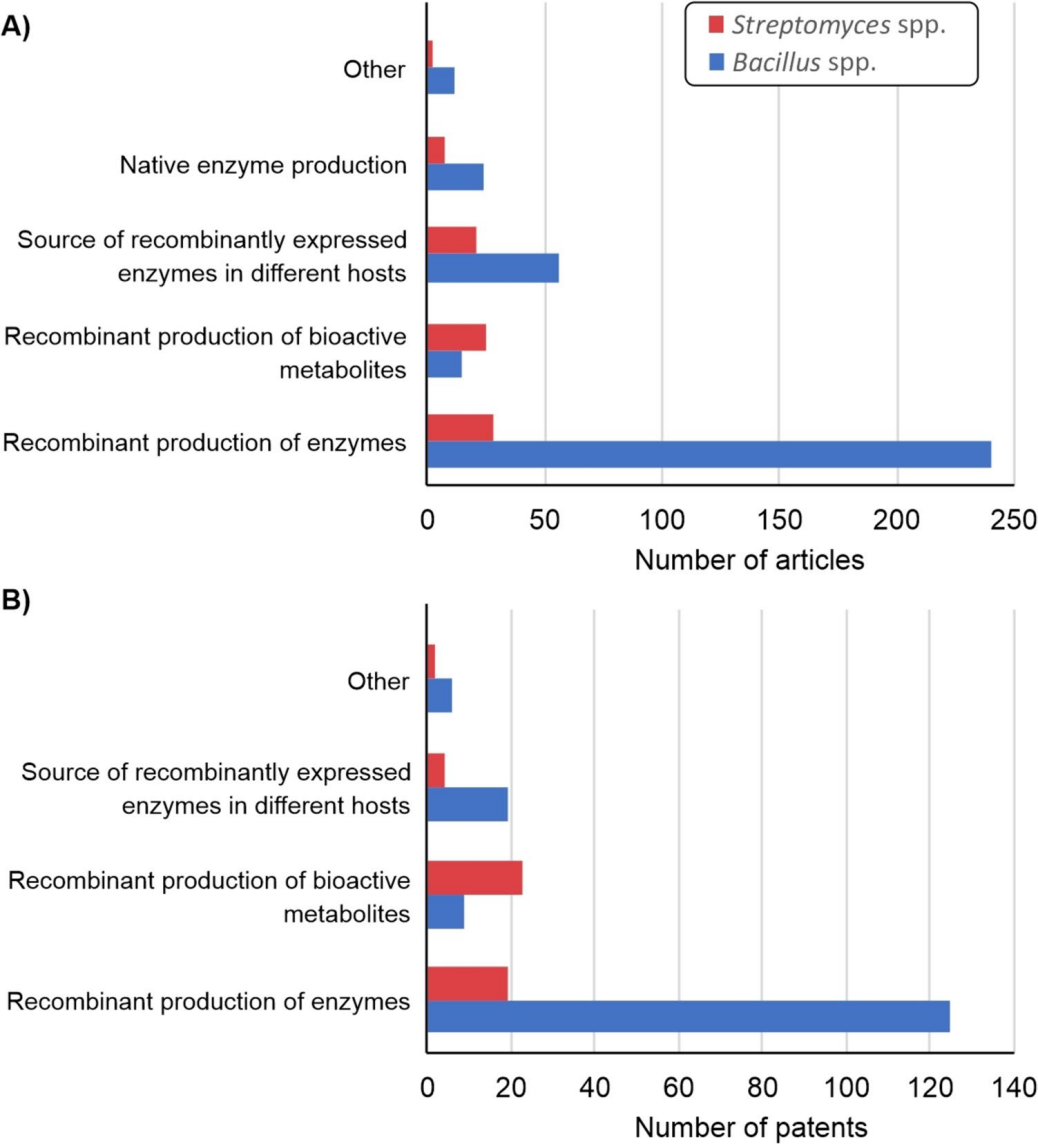
Distribution of the results obtained from SciFinder database (accessed on 22 April, 2023) with the following query: (recombinant [Abstract/Keywords]) AND (enzyme [Abstract/Keywords]) AND (industrial strain [Abstract/Keywords]) AND (Genus (*Bacillus* or *Streptomyces*) [Title]) NOT (*E. coli* [Abstract/Keywords]) (period 2000-present), then manually checked and integrated. Distribution of results related to *Bacillus* and *Streptomyces* genera presented in the journals (**A**) and patents (**B**)

In order to analyze the literature data since year 2000 regarding *Bacillus* or *Streptomyces* spp. as host or source of industrially relevant products, search was performed within the SciFinder, a comprehensive database for the chemical literature including patents. The main results obtained from this analysis are highlighted in Fig. 1. Using the same criteria for searching, 347 scientific papers related to genus *Bacillus* were selected, comparing to only 84 scientific papers related to genus *Streptomyces*.

Out of 347 scientific papers, nearly 70% were about recombinant enzymes produced in different *Bacillus* spp. as hosts, whereas in the case of *Streptomyces*, this percentage was only 33%, indicating that a considerably smaller proportion were associated with production of recombinant enzymes in different *Streptomyces* spp. It is worth mentioning that almost the same percent of scientific articles related to *Streptomyces* hosts, 30% out of 84, is describing production of bioactive secondary metabolites, reflecting the traditional exploitation of these bacteria for production of valuable bioactive molecules used in pharmaceutical, agricultural, and food industries, also suggesting their robustness and scalability of cultivation. On the other side, in relative comparison, there is 1.5-fold more scientific papers describing *Streptomyces* spp. as source of recombinantly produced enzymes in comparison to *Bacillus* spp., while there is a comparable level of studies dealing with native protein production in these hosts (about 10%) (Fig. 1A). The same search of patents identified 159 and 48 patents connected to *Bacillus* and *Streptomyces* spp., respectively (Fig. 1B). The distribution of results among patents enlisted in our search through SciFinder database was similar to those obtained for scientific papers. Among the results related to *Bacillus* as host, 78% (125 out of 159 patents) was focused on the optimal expression of genes for various enzymes produced in *Bacillus*, comparing to 40% (19 out of 48 patents) related to genus *Streptomyces*. Moreover, 48% of *Streptomyces*-related patents were actually describing the production of bioactive secondary metabolites in *Streptomyces* hosts. In the relative comparison, there were 1.5-fold more patents describing *Bacillus* spp. as source of recombinantly produced enzymes in comparison to *Streptomyces* spp. (Fig. 1B).

Given the innate capability of efficient protein secretion, both genera were considered for catalogue expansion of the microbial cell factories (Ferrer-Miralles and Villaverde 2013). Secretion of recombinant proteins contributes to reduced toxicity to host cells, promotes correct folding, and makes the downstream recovery an easier task (Anne et al. 2017). Among Gram-positive bacteria employed for producing industrially relevant enzymes, *Bacillus* species stand as the primary choice for recombinant host microorganisms (Liu et al. 2013). They possess commendable genetic tractability, demonstrate robust growth in laboratory settings, and benefit from a wide array of genetic tools and expression vectors, making them exceptionally suitable for genetic modifications and cultivation. Furthermore, the majority of *Bacillus* strains do not have apparent pathogenicity; they are considered as GRAS (generally recognized as safe) organisms, so there is no need to perform costly processes for the removal of toxic impurities such are lipopolysaccharides (LPS) or endotoxins. Various *Bacillus* spp. possess significant fermentation capacity, making them ideal for large-scale production of industrially important proteins, sometimes in gram quantities per liter. The streptomycetes are also recognized for their robustness and scalability as industrial strains thanks to their long-lasting usage as antibiotic producers with novel genes for both the biosynthesis and the improvement of target molecules production being identified (Ndlovu et al. 2015; Xu et al. 2022). Furthermore, streptomycetes exhibit low endogenous proteolytic activity, rapid and cost-effective growth in inexpensive media, non-pathogenic characteristics, and absence of pyrogenic LPS and endotoxin production, but also the ability to express G/C-rich genes without the need to optimize codon usage (Anne et al. 2012; Sevillano et al. 2013). The extensive fermentation expertise gained from industrial antibiotic production, combined with the availability of a diverse array of genetic manipulation tools, has led to streptomycetes gaining recognition as an excellent host for the production of industrial enzymes (Berini et al. 2020).

## Molecular tools developed for increased production of recombinant enzymes in *Bacillus* and *Streptomyces* spp.

Protein production is a complex task, and its optimization involves influencing various stages and adjusting different parameters, often with simultaneous optimization of expression elements and host strain optimization. Noteworthy are outstanding mini-reviews covering key parameters influencing production of recombinant proteins in Bacillus spp. (Pohl and Harwood 2010; Liu et al. 2013; van Dijl and Hecker 2013; Yang et al. 2021) and Streptomyces spp.(Berini et al. 2020). In addition to review articles that specifically delve into engineering either Bacillus or Streptomyces hosts for the efficient production of heterologous proteins, there are also papers that explore the optimization of specific expression elements, such as signal peptides, in bacterial expression systems (Freudl 2018; Cai et al. 2019; Hwang et al. 2021).

### Promoter optimizations

One of the most cost-effective and efficient methods to achieve high-level production of recombinant proteins remains the optimization of the promoter at the level of transcription. Transcriptomic data are often used for the identification of strong promoters, either native or heterologous, and promoter engineering, including semi-rational evolution, is a powerful tool for increasing the target protein production (Miao et al. 2020). Some of the promoters used for recombinant production of industrially relevant enzymes in *Bacillus* and *Streptomyces* spp. are listed in Tables 1 and 2, respectively. It is the common feature in both expression systems to use strong constitutive promoters like promoter of *aprE* gene encoding subtilisin, a *Bacillus subtilis* extracellular proteolytic enzyme, P_43_ promoter from *B. subtilis* gene encoding cytidine deaminase, or several promoters of different *amy* genes coding for alpha-amylase in various *Bacillus* spp. Concerning *Streptomyces* hosts, strong constitutive promoters like P_*pstS*_ promoter of gene coding for phosphate-binding protein in *Streptomyces lividans*, promoter of *Streptomyces venezuelae* subtilisin inhibitor gene vsi, or the promoter region of the erythromycin resistance gene (P_*ermE*_) of *Saccharopolyspora erythraea* are being used. Still, to control gene expression for many industrially relevant enzymes in order to avoid their toxicity, inducible promoters are regularly used, despite the inevitable increase in production costs. As an illustration, high-yield production of levansucrase was obtained when *Bacillus megaterium* was cultivated in a medium utilizing raw glycerol from the biodiesel industry as a carbon source. This was achieved by employing the xylose-inducible *xylA* promoter, pointing towards the potential application of this system in existing biorefinery concepts (Korneli et al. 2013). Xylose was also used to induce expression of gene for *Thermus thermophilus* alkaline phosphatase in *S. lividans*, driven by P_*xysA*_ promoter from *Streptomyces halstedii* (Diaz et al. 2008). In *Streptomyces* hosts, the genetic tools previously used for years for synthesis of secondary metabolites are frequently utilized, including promoters induced by thiostrepton (*tipA* promoter involved in autogenous transcriptional activation of the *tipA* gene by thiopeptides) or tetracycline (*tcp830*, the synthetic thiostrepton controllable promoter active in a wide range of *Streptomyces* species) (Rodríguez-García et al. 2005). The improved recombinant protein production in both *Bacillus* and *Streptomyces* hosts is often accomplished using tandem promoters, so the triple-promoter was efficient for obtaining alleviated production of nattokinase in *B. subtilis* (Table 1) while dual-promoter was used for recombinant production of phospholipase D in *Streptomyces lividans* (Table 2) (Liu et al. 2019c; Tao et al. 2019). Recently published patent is based on production of the collagenase by *B. subtilis* WB600 strain, with the synthetized collagenase gene expressed from dual promoter P_*HpaII-p43*_ (Zhang et al. 2022).

**Table 1:** Representative recombinant enzymes produced in *Bacillus* spp.

Protein	Source	Host	Plasmid	Promoter	Inducer	Signal peptide	Activity	References
Keratinase	Soil metagenome	*B. subtilis* WB600	pMA5	*aprE*	-	native	2605 U mL^−1^	Gong et al. (2020)
Nattokinase	*B. subtilis* natto	*B. subtilis* WB800	pMA0911	*HpaII*-*HpaII*-*p43* tandem	-	WapA	264 U mL^−1^	Liu et al. (2019c)
Neutral protease	*B. amyloliquefaciens* K11	*B. amyloliquefaciens* K11	pUB110	native	-	native	7460 U mL^−1^	Wang et al. (2016a)
Alkaline protease	*B. clausii*	*B. amyloliquefaciens* K11	pUB110	*amyQ*	-	AprE	13,800 U mL^−1^	Wang et al. (2019a)
Serine alkaline protease	*Idiomarina* sp. C9-1	*B. subtilis* WB600	pMA5	*aprE*	-	LipB	4935 U mL^−1^	Zhou et al. (2018)
Cellulases	*Bacillus* sp. KSM-S237	*B. subtilis* ISW1214	pHYEglS	native	-	native	50 U mg^−1^	Hakamada et al. (2000)
α-Amylase	*B. stearothermophilus*	*B. subtilis* WHS11YSA	pHYCGTd4	*HpaII*-*amyQ*, dual	-	YojL	1497 U mL^−1^	Yao et al. (2019)
α-Amylase	*Pyrococcus furiosus*	*B. amyloliquefaciens*	pUBC19-PamyA-*mpfa*	*amyA*	-	AmyA	2714 U mL^−1^	Wang et al. (2016b)
Chitinase	*Bacillus* sp. DAU101	*B. subtilis* WB600	pP43NMK	*p43*	-	NprB	52 U mL^−1^	Pan et al. (2019)
Chitosanase	*B. subtilis* 168	*B. subtilis* PT5	Integration vector pMT1-Csn	native	-	AprE	156 U mL^−1^	Su et al. (2017)
Pullulanase	*B. naganoensis*	*B. subtilis* WB600	pMA0911	*sacB*	sucrose	LipA	26 U mL^−1^	Deng et al. (2018)
Pullulanase	*B. naganoensis*	*B. subtilis* ATCC6051∆10	pBE-MCS	*amyL*–*spovG* dual	-	native	626 U mL^−1^	Liu et al. (2018)
Lipase	*Proteus vulgaris* T6	*B. subtilis* WB800	pHPQ-PVL	*sacB*; *p43*-*degQ* cassette	sucrose	SacB	357 U mL^−1^	Lu et al. (2010)
Lipase A	*B. subtilis* 168	*B. subtilis* BNA with co-overexpressed *secDF*	pHP13L	P_AE_	NA	NA	288 U mL^−1^	Ma et al. (2018)
Phospholipase D	*Streptomyces racemochromogenes*	*B. subtilis* WB600	pMA0911-PLD-amyE-his	*HpaII*	-	AmyE	24 U mL^−1^	Huang et al. (2018)
Inositol monophosphatase	*Thermotoga maritima*	*B. subtilis* SCK22	pHT7-IMP	T7-*lac* hybrid	IPTG	native	NA-not available	Ye et al. (2022)

*NA* not available

**Table 2:** Representative recombinant enzymes produced by *Streptomyces* spp

Protein	Source	Host	Plasmid	Promoter	Inducer	Signal peptide	Activity	References
Pernisine	*Aeropyrum pernix*	*S. rimosus*	pVFPER5	*tcp830*	-	SrT	NA	Šnajder et al. (2019)
Keratinase	*Streptomyces* sp. SCUT-3	*Streptomyces* sp. SCUT-3	pSET-*sep39*	Native	-	Native	64 U mL^−1^	Li et al. (2020)
Amylases	*S. griseus* IMRU3570	*S. lividans ∆TA-Tox* toxin	pNRoxAnti-Amy	*pstS*	-	PstSP	1100 U mL^−1^	Sevillano et al. (2017)
Xylanase	*S. halstedii* JM8	*S.lividans ∆TA-Tox* toxin	pNRoxAnti-Xyl	*pstS*	-	PstSP	170 U mL^−1^	Sevillano et al. (2017)
	*Aspergillus nidulans*	*S. lividans* JI66	pTXA3 (pIJ702 derivative)	*pxysA*	Xylose	SpS	19 U mL^−1^	Díaz et al. (2004)
Cellulase	*Rhodothermus* *marinus*	*S. lividans* TK24	pIJ486-*vsi*-*celA*	*vsi*	-	VSI	15 U mg^−1^	Hamed et al. (2017)
β-Glucosidase	*Thermobifida fusca* YX	*S. lividans* 1326	pUC702-Tfu1074	*pldp*	-	PLD SP	4200 U mL^−1^	Noda et al. (2010)
Chitinases	*Streptomyces coelicolor* A3(2)	*S. lividans* 10–164 [msiK^−^]	pC109-*chiC* (derived from pIJ702)	NA	NA	NA	9215 U mg^−1^	Nguyen-Thi and Doucet (2016)
	Metagenomic	*S. coelicolor* A3(2)	pIJ86	ermE*	-	Native	18.5 U g^−1^	Berini et al. (2019)
Chitosanases	*Kitasatospora* sp. N106	*S. lividans* Δ*csnR*	pHM8aBΔM	ermE*	-	NA	24 U mL^−1^	Dubeau et al. (2011)
Lipase	Metagenomic	*S. lividans* *msiK*^−^	pIAFD95A	*D95A*	NA	NA	160 U mg^−1^	Meilleur et al. (2009)
Phospholipase								
	*Streptoverticillium cinnamoneum*	*S. lividans* 1326	pUC702-PLD	Native	-	Native	20,000 U L^−1^	Ogino et al. (2004)
	*Streptomyces racemochromogenes*	*S. lividans* TK23	pES103	Native	-	Native	30 U mL^−1^	Nakazawa et al. (2011)
	*Streptomyces halstedii*	*S. lividans* TK24	pIJ12739-PLD	Induced (*tip*)/const (ermE*) dual	Thiostrepton	Native	68 U mL^−1^	Tao et al. (2019)
	*Streptomyces antibioticus*	*S. lividans* SBT5	pOJ260-*rep*3-pld*	kasO*	-	*sigcin*	87 U mL^−1^	Wang et al. (2022)
	*Streptomyces* sp. NA684	*S. lividans* 1326	pUC702-PLB	*pld*	-	PLD SP	14 U mL^−1^	Matsumoto et al. (2013)
Alkaline phosphatase	*Thermus thermophilus*	*S. lividans* JI66	pTXF1 (pIJ702 based)	*xysA*	Xylose	Native	267 U mL^−1^	Diaz et al. (2008)
Phytase	*E. coli*	*S. rimosus*	pVF (pVF-P*tcp830*- *srT*-*appA*)	tcp830*	Tetracycline	*srT*	5 U mL^−1^	Carrillo Rincón et al. (2018)
Transglutaminase	*Streptomyces hygroscopicus*	*S. lividans* TK24	pTGO (pIJ86 based)	Native Optimized	-	Native	2 U mL^−1^	Liu et al. (2016)

*NA* not available

### T7-like expression systems

In both *Bacillus* and *Streptomyces* hosts, attempts were made to obtain the system that could mimic the well-known *E. coli* T7 polymerase–driven expression system. Using a novel *B. subtilis* strain SCK22 with T7 RNA polymerase gene inserted into the chromosome, several recombinant proteins were successfully produced. The plasmid called pHT7 was utilized, containing various recombinant proteins, including α-glucan phosphorylase, inositol monophosphatase, and 4-α-glucanotransferase. The expression was under tight control of the T7 promoter, which was accompanied by a ribosome binding site (RBS) specific to *B. subtilis*. The results were highly promising, with excellent expression levels achieved, accounting for approximately 25–40% relative to the total proteins in cell (Ye et al. 2022). Likewise, a codon-optimized gene for T7 RNA polymerase was successfully integrated into the chromosome of another strain, *S. lividans* 10–164. The gene expression was regulated by the thiostrepton-inducible *tipA* promoter. Finally, a shortened *S. lividans* xylanase A was recombinantly produced using the T7 promoter in this setup (Lussier et al. 2010).

### Control at translation level

At the translation level, careful selection of appropriate RBS and signal peptides (SP) has significantly contributed to achieving high yields of protein production. The SP serves as a crucial regulatory element located at the N-terminus of the target protein. Its function is essential for proper directing to the translocation system and subsequent export into the growth medium. To attain high-level production of desired enzyme, selecting an appropriate SP regulatory element is of utmost importance. In cases where native SPs are either absent or non-functional, heterologous genes are often fused with SP-encoding sequences sourced from genes for highly produced/secreted endogenous proteins, either in *Bacillus* or *Streptomyces* hosts (Tables 1 and 2).

In both *Bacillus* and *Streptomyces* species, the majority of protein exports are facilitated through the general secretory (Sec) pathway. Alternatively, a set of more specialized transport systems, including the twin-arginine translocation (TAT) pathway, is also available for protein export (Anne et al. 2014). Using genetic engineering, the existing modalities for protein export could be modified in a way that secretion of heterologous proteins is facilitated. By solely modifying the SecA-dependent secretion pathway, the productivity of alkaliphilic thermostable alkaline cellulase (Egl-237) in *B. subtilis* was doubled (Kakeshita et al. 2010). Moreover, by manipulating the secretion pathway through deletion of the *sipY* gene, which encodes a major signal peptidase, the production of *S. coelicolor* agarase in *S. lividans* was significantly enhanced (Gabarró et al. 2017).

### Host optimizations through genome editing

Intracellular and extracellular proteases produced by *Bacillus* host could be important obstacle for efficient secretion of recombinant proteins; hence, protease-deficient *Bacillus* strains, genetically engineered to circumvent host-mediated proteolysis, were made. *B. subtilis* derivative WB600, lacking six extracellular proteases, demonstrated increased productivity of several recombinant proteins, as well as WB800, which lacks eight extracellular proteases (Table 1). The same strategy was used to generate *B. subtilis* WS11 strain with 11 proteases deleted from its genome (Zhang et al. 2018).

Over the past few decades, the emergence of multi-omics technology has facilitated the development of genome-reduced microbial strains that surpass their wild-type counterparts related to target protein productivity (Reuss et al. 2017). The mini *Bacillus* strain PG10, which has approximately 36% of the *B. subtilis* genome deleted, has shown potential for recombinant production of “difficult proteins.” Specifically, it successfully produced four different staphylococcal antigens that were challenging to produce using the currently applied *B. subtilis* strains (Aguilar Suárez et al. 2019). While mini *Bacillus* strains require further optimization to address concerns related to product degradation, reduced cell lysis, and their feasibility for large-scale fermentation, the concept of genomic streamlining as a way to create future *Bacillus* cell factories remains an incredibly appealing strategy. Similarly, there is a continuous expansion in the number of genome-reduced industrial *Streptomyces* chassis, which serves as excellent hosts for heterologous production of secondary metabolites and recombinant proteins (Bu et al. 2019; Gren et al. 2021; Hwang et al. 2021). An example is industrial strain *Streptomyces rimosus* in which one of the highest titer records for heterologous antibiotics production was achieved, represented in production of several grams per liter for chlortetracycline (Wang et al. 2019b). Also, when 15 biosynthetic gene clusters were deleted in the chromosome of *Streptomyces albus* Del14, reducing the genome by 500 kb (7.3% of the entire genome), an ideal *Streptomyces* chassis with improved compound detection limit was obtained (Myronovskyi et al. 2018).

It is noteworthy that *Bacillus* spp. could serve as adequate hosts for difficult-to-express eukaryotic genes for model proteins. To achieve this, an efficient *B. subtilis* expression toolbox was developed, comprising a collection of 60 expression vectors. Numerous combinations of two promoter variants, four potent secretion signals, a downstream box for enhancing translation, and three plasmid backbones were designed in these vectors. This toolbox was successfully employed for the overproduction and secretion of sulfhydryl oxidase Sox from *Saccharomyces cerevisiae* and the human interleukin-1β. The expression was carried out in a tailor-made, protease- and sporulation-deficient *B. subtilis* strain with reduced autolysis and secondary metabolism (Krüger et al. 2022).

### CRISPR/Cas systems

In the field of industrial biotechnology, several genome editing techniques have proven successful in meeting the increasing demands and expanding the range of chemicals, metabolites, and biomolecules produced by microbes. The clustered regularly interspaced short palindrome repeat (CRISPR)/CRISPR-associated protein (Cas) systems played a pivotal role in achieving these objectives (Donohoue et al. 2018). Originally identified as microbial adaptive immune systems used to combat invading mobile genetic elements, CRISPR/Cas systems have been repurposed to enable a wide array of genetic modifications in various species relevant to industrial biotechnology and among them both *Bacillus* and *Streptomyces* spp. The application of a type II CRISPR-Cas9 system from *Streptococcus pyogenes* proved successful in *B. subtilis* as a single-plasmid system. This approach enabled efficient genome editing, demonstrated by deleting two large regions in the *B. subtilis* chromosome and repairing the *trpC2* mutation, known to be a non-reverting 3-bp deletion of *B. subtilis* 168, tryptophan-requiring auxotroph used in many industrial processes (Altenbuchner 2016). Also, CRISPR-Cas9 system for *B. subtilis* was used for in situ modification of the *aprE* gene (Price et al. 2019). This gene encodes subtilisin E, an enzyme which finds widespread use in the detergent industry worldwide. Using the CRISPR-Cas9 system, a sequence for salt-bridge triad (Arg19-Glu271-Arg275), identified in M-protease from *Bacillus clausii* and responsible for the enzyme’s characteristic thermotolerance, was successfully introduced into the *aprE* gene, resulting in increased thermotolerance and activity of subtilisin E. The application of integrative plasmids through CRISPR-Cas9 technology proved to be a feasible and effective approach for producing recombinant phytase in *B. subtilis*. This method resulted in the creation of a stable strain with minimal risk of horizontal transfer of the engineered traits (Santos et al. 2019). To knock out the gene encoding stage II sporulation protein AC, *spoIIAC*, in *B. subtilis* KO7, Fragment Exchange (FX) plasmid tools for CRISPR/Cas9-mediated gene integration were employed (García-Moyano et al. 2020). The *B. subtilis* KO7 serves as a model strain for genetic engineering, primarily due to its low protease background resulting from the deletion of seven protease-encoding genes. By utilizing the asporogenic *Bacillus* host, the risk of cross-contamination can be effectively minimized in fermentations performed in both laboratory and industrial settings. By employing the double-plasmid system for CRISPR-Cas9 editing, the researchers successfully integrated the *B. licheniformis aprE* gene into the chromosome of the asporogenic *B. subtilis* KO7S2 strain, obtaining the stable production of subtilisin. The challenges and progress in genome editing technologies in *Streptomyces* spp. are already well described, but almost exclusively related to streptomycetes as producers of secondary metabolites (Alberti and Corre 2019; Zhao et al. 2020). Using bioinformatic analysis of sequenced *Streptomyces* genomes, researchers have discovered the existence of silent biosynthetic gene clusters (BGCs). These clusters represent unexplored and abundant reservoirs of natural compounds, offering promising prospects for the discovery of novel chemical compounds. An efficient CRISPR-Cas9 knock-in strategy was employed to activate multiple BGCs of various classes in five *Streptomyces* species. This approach successfully induced the production of unique metabolites, among which was a novel pentangular type II polyketide discovered in *S. viridochromogenes* (Zhang et al. 2017). Also, the type II CRISPR/Cas system from *S. pyogenes* was effectively reconstituted in three different *Streptomyces* species, allowing for targeted multiplex genome editing, including chromosomal deletions ranging from 20 bp to 30 kb. Remarkably, the editing efficiency achieved ranged from 70 to 100% (Cobb et al. 2015). Nowadays, for both *Bacillus* and for *Streptomyces* spp., many second-generation CRISPR systems have been developed (e.g., base editors) enabling directed evolution of special proteins in *B. subtilis* or single-nucleotide-resolution genome editing without requiring a DNA double-strand breaks in *Streptomyces* spp. (Hao et al. 2022; Tong et al. 2019).

### Spore display

In the field of biotechnology, there is a growing interest in utilizing spore display for heterologous proteins anchored on spores. This approach aims to enable better functionality and stability of recombinant proteins, opening up new possibilities for various applications (Zhang et al. 2020). Typically, the gene encoding a target protein is fused with a gene for the spore coat protein (Cot) in actively growing *B. subtilis* cells. Gene for Tm1350 lipase, derived from the hyperthermophilic bacterium *Thermotoga maritima* MSB8, could serve as an example since it was successfully expressed and displayed on the *B. subtilis* spores, anchored to CotB (Chen et al. 2015). Another example is tyrosinase from *B. megaterium*, effectively displayed on *B. subtilis* spores anchored to CotE (Hosseini-Abari et al. 2016). By the way, this tyrosinase was intended to be used for the production of levodopa (L-DOPA) from l-tyrosine as a treatment for Parkinson’s disease. There is also a potential industrial application for β-galactosidase from *E. coli*, extensively employed to enhance the sweetness, solubility, and digestibility of dairy products or for a mutant trehalose synthase (V407M/K490L/R680E TreS), originally isolated from *Pseudomonas putida* and utilized in the production of trehalose that serves as a food additive. Both enzymes are displayed on the surface of *B. subtilis* spores with either CotE and CotG or CotC and CotG as anchoring motives (Hwang et al. 2013; Liu et al. 2019a). Besides production of valuable industrial commodities, enzymes displayed on *Bacillus* spores have found utility in agriculture as eco-friendly biopesticide (Rostami et al. 2017).

### Biocatalysts

Both *Bacillus* or *Streptomyces* spp. produce various enzymes used in biotransformation processes, preferable alternative to classical chemical-based transformation of substrates if we have in mind increasing environmental concerns (Wachtmeister and Rother 2016; Anteneh and Franco 2019). In order to overcome limitations like restricted enzyme stability, cofactor requirements, and susceptibility to changes in operating conditions, whole-cell *Bacillus* and *Streptomyces* biocatalysts are extensively used. Likewise, employing biocatalysts as whole cells eliminates the requirement for cell lysis and enzyme purification, resulting in significant cost savings. Achieving regioselective oxidations with synthetic organic chemistry can be challenging so cytochrome P450 enzymes (P450s) are used instead. Several papers are describing the usage of *B. megaterium* as a host for whole-cell biotransformation with recombinant P450 enzymes, applied in pharmaceutical industry (Kleser et al. 2012; Kiss et al. 2015; König et al. 2019; Richards et al. 2020). For example, Richards et al. (2020) explored different approaches to overcome limitations for whole-cell N-demethylation of noscapine with P450_BM3_ (CYP102A1) mutant enzyme recombinantly produced in *B. megaterium* using xylose-inducible promoter P_*xylA*_*.* Besides medical, cell surface display technology is frequently used with *Bacillus* hosts producing enzymes for environmental and industrial application (Chen et al. 2017). The majority of textile dyes are hazardous, being toxic, carcinogenic, or mutagenic to humans. Additionally, they are relatively resistant to degradation, posing a significant environmental threat in a form of waste from textile industry. Microbial or enzymatic decolorization and degradation are considered highly attractive approaches to address this issue due to their cost-effectiveness and minimal environmental impact. Efficient decolorization of textile dye RB19n with *B. thuringiensis* cells displaying mutant laccase from *Shigella dysenteriae*, immobilized on volcanic rock matrix, is one example of this methodology (Wan et al. 2017). The gene for WlacD laccase was expressed from P_*cry3Aa*_ promoter, modified version of *cry* promoter, responsible for the overproduction of crystal proteins in *B. thuringiensis*. The P_*cry3Aa*_ promoter was generated when -35 and -10 sequences of *cry* promoter were replaced with the consensus sequences of *σ*^*A*^-dependent promoter of *B. subtilis*. Two repeat N-terminal domains of autolysin Mbg (Mbgn)_2_ were used as the anchoring motif, and whole-cell laccase biocatalyst was finally prepared when *B. thuringiensis* cells were adsorbed by volcanic rock matrix. The identical anchoring motif was utilized to develop a chitinolytic whole-cell *B. thuringiensis* biocatalyst. (Tang et al. 2017). This biocatalyst displayed enhanced catalytic and antifungal activities.

*Streptomyces* spp. possess a versatile metabolism, making them valuable sources of biocatalytic tools for various innovative biotechnological applications. Depolymerases, laccases, and pectinases are commonly utilized for biomass degradation or bioremediation, whereas tyrosinases, lipases, and polygalacturonases find extensive use in the pharmaceutical industry (Spasic et al. 2018). In our previous work, we have identified novel and functional biocatalysts from selected *Streptomyces* spp. when we applied a combination of conventional microbiological and biochemical screens, along with genome sequencing and analysis (Ferrandi et al. 2021). Besides their valuable part in biotechnological production processes of drugs, whole-cell *Streptomyces* spp. as biocatalysts play an important role in biotransformation of diverse substrates, with the ultimate goal to obtain more potent and less toxic compounds, as well as biofuels (Anteneh and Franco 2019; Barbuto Ferraiuolo et al. 2021). Class I P450 enzymes from *Actinomycetes*, CYP107B1 and CYP105D1, were produced in *S. lividans* using P_*tipA*_ promoter, and their efficiency in whole-cell biotransformation assay with 7-ethoxycoumarin subjected to oxidative dealkylation was proved (Ueno et al. 2005). The same host was exploited for recombinant expression of gene for another cytochrome P450 enzyme, P450Rhf from *Rhodococcus* sp. NCIMB 9784, using P_*nitA*_-NitR hyper-inducible expression system for *Streptomycetes*, and the efficiency of this whole-cell biotransformation was also confirmed (Ueno et al. 2014). Whole *Streptomyces* cells are capable of catalyzing a wide range of oxidative-type reactions (Salama et al. 2022). A cytochrome P450 gene from *Streptomyces ahygroscopicus* ZB01 was recombinantly expressed in *S. lividans* and used in whole-cell biocatalytic assay for regiospecific conversion of avermectin into 4″-oxo-avermectin (Jiang et al. 2012). Biotransformation of daidzein was accomplished with several recombinant isoflavone O-methyltransferases from *Streptomyces* spp. when their genes were expressed in *S. avermitilis* ΔSaOMT2, under the control of constitutive *ermE* promoter (Choi et al. 2013).

## Industrially relevant enzymes produced in *Bacillus* spp.

It is estimated that *B. subtilis*, *B. amyloliquefaciens*, and *B. licheniformis* collectively contribute to approximately 50% of the global industrial enzyme production. These enzymes include proteases, α-amylases, β-glucanases, and penicillin acylases. Some of the most relevant recombinant industrial enzymes produced in *Bacillus* spp. are summarized in Table 1. Important elements that can influence the level of gene expression and the overall productivity of active enzymes, including promoters and signal peptides, are also listed.

### Proteases/peptidases

**Keratinases.** Keratinolytic enzymes show great potential for diverse industrial applications. Their ability to break down resilient keratinous materials enables the production of more valuable products and offers a favorable alternative to conventional physicochemical treatments. Keratinous materials in the form of agro-industrial wastes as readily available substrates now offer opportunities for the production of high-value products. The common strategy to evaluate the efficiency of several promoters in order to select the best one was applied with the keratinase gene expressed in *Bacillus subtilis* WB600 when the *aprE* promoter was selected as superior candidate among 10 evaluated (Gong et al. 2020). Specially, among the 10 promoters tested, a significant promotion of keratinase activity from 165 U mL^−1^ to 2605 U mL^−1^ was obtained when *aprE* promoter was used. *B. subtilis* derivative WB600, deficient in six extracellular proteases, yielded higher productivity of keratinase. Using the batch fermentation mode, keratinase activity was further improved to 7176 U mL^−1^, and when the fed-batch fermentation mode was applied, the maximal activity up to 16860 U mL^−1^ was obtained. The up scaling of production in the fermenter resulted in the highest keratinase activity, which was subsequently proven effective in feather degradation. This breakthrough creates an opportunity for utilizing this enzyme to control the accumulation of feather wastes in environmentally friendly manner. A recent patent application is in fact describing production of recombinant keratinase in *B. subtilis* (You et al. 2022).

**Nattokinase**. Nattokinase, a member of the subtilisin family, is recognized as a nutraceutical for cardiovascular conditions due to its powerful fibrinolytic capabilities. There is a currently active patent based on production of nattokinase by food-grade *Bacillus licheniformis*, with the expression level significantly increased when multiple copies of nattokinase gene were integrated into the genome, resulting in enzyme titer of 1.45 g L^−1^ (Wang et al. 2016c).

**Neutral proteases.** Many genes for neutral proteases were cloned from various *Bacillus* spp. and expressed either in homo- or heterologous *Bacillus* hosts. The activity of neutral protease produced by *B. subtilis* strain AS.1398, an industrial strain used in China for decades, reached 5500–7000 U mL^−1^ (Li et al. 2013). This strain was developed through traditional mutation breeding techniques, which involved UV treatment, chemical mutagenesis, and further optimization of fermentation parameters and medium. In comparison to *B. subtilis*, *B. amyloliquefaciens* exhibits robust secretory ability, but it only produces a limited amount of its own secretory proteins (Wang et al. 2019a). Nevertheless, *B. amyloliquefaciens* K11 appeared as a suitable candidate for economic production and industrial applications of recombinant neutral protease with activity of 7460 U mL^−1^ (Table 1), reaching four times higher increased activity of 28084 U mL^−1^ by process optimization (Wang et al. 2016a).

**Alkaline proteases**. Alkaline protease from *B. clausii* (*Bc*aprE) was produced in *B. amyloliquefaciens* reaching activity of 13,800 U mL^−1^ (Table 1). Knocking out the endogenous neutral protease-encoding gene *Banpr* resulted in further activity improvement by 25.4%. Finally, the titer of *Bc*aprE enzyme was scaled up to 20–30 g L^−1^ through fermentation optimizations (Wang et al. 2019a).

### Glycosyl hydrolases

**Cellulases**. As a part of the search for thermostable cellulases that could be used as additives for improving the cleaning effect in detergents, several recombinant extracellular cellulases from the anaerobic bacterium *Clostridium thermocellum* were produced in *B. subtilis* in combinations with 173 different *B. subtilis* signal peptides (Lan Thanh Bien et al. 2014). Thermostable alkaline cellulase Egl-237 from *Bacillus* sp. strain KSM-S237 was produced in *B. subtilis* yielding high carboxymethyl cellulase titer of 2.0 g L^−1^ (Hakamada et al. 2000).

**α-Amylases**. There are limitations to obtain the high amount of α-amylase in *B. subtilis* mainly because of the secretion stress (Yan and Wu 2017). The high-level production of *B. stearothermophilus* AmyS in *B. subtilis* was successfully accomplished through a combination of strategies. This involved screening for optimal signal peptides, strategy to overexpress chaperone genes, and to randomly mutate the amylase gene (Yao et al. 2019). This approach resulted in the activity of 1497 U mL^−1^ (Table 1), which was further enhanced in 3-L fermenter reaching 9201 U mL^−1^, corresponding to the highest activity reported for extracellular *B. stearothermophilus* AmyS in *B. subtilis*. Also, the α-amylase from *Pyrococcus furiosus*, which holds significant potential for industrial starch processing due to its thermostability, extended half-life, and optimal activity at low pH was effectively produced in *B. amyloliquefaciens* resulting in the activity of 2714 U mL^−1^ (Wang et al. 2016b). The quest for highly efficient α- amylase is continuing, with the activity of 2974 U mL^−1^ reached when AmyZ1 from a deep-sea bacterium *Pontibacillus* sp. ZY was recombinantly produced in *B. subtilis*, described in patent field by Anhui University in China (Xiao et al. 2022).

**Chitinases and chitosanases**. Glycosyl hydrolases that catalyze the hydrolysis of β-1,4-glycosidic bonds of chitin and its deacetylated derivative, chitosan, are gaining increasing attention. Their potential application lies in the bioconversion of chitinous biowaste into products of significant commercial value, with relevance in medicine, agriculture, cosmetics industries, and as food additives. Combination of various strategies based on genetic engineering resulted in 52 U mL^−1^ activity of *Bacillus* chitinase in *Bacillus subtilis* WB600 (Table 1). The chitosanase encoded by the *B. subtilis* 168 *csn* gene was recombinantly produced in *B. subtilis* PT5 (Su et al. 2017). The production of recombinant protein was improved by using the AprE signal peptide instead of the original SP, reaching the activity of 156 U mL^−1^ (Table 1). The highest achieved activity of Csn protein, produced using the optimal fermentation medium, was 208 U mL^−1^ in a 5-l fermenter.

**Pullulanases**. Thermoduric pullulanases, functioning as starch-debranching enzymes, play a key role in the production of concentrated glucose, maltose, and fructose syrups used in the food and beverage industries. Furthermore, pullulanases are included in detergent formulations, in combination with other enzymes, to effectively remove starch-based stains. In both cases, these enzymes are used at specific conditions, mainly at temperatures above 60 °C and at pH 4.5, implying high thermostability and activity preserved at defined pH range. The optimization of extracellular thermoduric pullulanase production, whether through their native hosts or by recombinant organisms, has been thoroughly documented (Akassou and Groleau 2019). Pullulanase from *Bacillus naganoensis* was produced in protease-deficient host strain *B. subtilis* WB800 under the control of inducible promoter whose activity was efficiently enhanced by regulating the *B. subtilis* WB800 DegQ enhancer, giving the activity of 26 U mL^−1^ (Deng et al. 2018). The productivity of the same enzyme was notably improved when the optimized *B. subtilis* strain was used as expression host (Liu et al. 2018). The ten-genes-deficient (genes for eight extracellular proteases, the sigma factor F and a surfactin) strain *B. subtilis* ATCC6051Δ10 was growing more rapidly and exhibited a remarkable capability to produce significantly higher amounts of extracellular protein when compared to the wild type. The highest activity of recombinant pullulanase (412.9 U mL^−1^) was reached when its gene was expressed downstream from the P_*spovG*_ promoter, promoter of septation protein SpoVG, using *B. subtilis* ATCC6051Δ10 as a host. The expression was further improved by using dual P_*amyL*_–P_*spoVG*_ promoter (promoter of α-amylase from *B. licheniformis* and promoter of septation protein SpoVG from *B. subtilis*, respectively) reaching maximum enzymatic activity of 626 U mL^−1^ (Table 1). The industrial applicability of this enzyme becomes evident from patents detailing the production of recombinant pullulanase in *B. amyloliquefaciens*, *B. subtilis*, and *B. licheniformis* (Wu et al. 2020; Liu et al. 2021; Niu et al. 2022). Notably, the latest patent from 2023 focuses on the production of recombinant pullulanase in a *B. subtilis* chassis that has been optimized using CRISPR-Cas technology (Chen et al. 2023).

**Lipases/esterases**. The large-scale industrial application of lipases and phospholipases, originally coming from *Bacillus*, *Pseudomonas*, and *Staphylococcus* species, is predominantly seen in the food and detergent industries. However, their utilization is rapidly expanding into biodiesel production, oil degumming, and the synthesis of flavor compounds and nutraceuticals. While *E. coli* is still the most widely used host for recombinant production of both lipases and phospholipases, the usage of *Bacillus* spp. as hosts remains limited. The large quantity of lipase from *Proteus vulgaris* was obtained when its gene was expressed from sucrose-inducible promoter in *B. subtilis* WB800 cells (Lu et al. 2010). The lipase activity in this system reached 357 U mL^−1^ (Table 1). On the other hand, the increased amount of extracellular lipase LipA from *B. subtilis* 168, produced in *B. subtilis* BNA, was obtained when the appropriate promoter was selected, i.e., expression system adjusted, and the selected components of secretion system overexpressed, resulting in activity of 288 U mL^−1^ (Ma et al. 2018). Interestingly, phospholipase D gene from *S. racemochromogenes* was successfully expressed in *B. subtilis* WB600 employing a range of combinatorial strategies, including SP screening, utilization of various plasmids, and optimization of the RBS and spacer region, reaching maximum activity of 24 U mL^−1^ (Table 1) (Huang et al. 2018). The patent application with previously described methodology for production of phospholipase D followed (Liu et al. 2019b). The usage of lipases as biocatalysts in biodiesel production is limited by issues related to their stability, reusability, and production costs. When the lipase A from *B. subtilis* was fused to crystal-forming protein Cry3Aa from *B. thuringiensis*, a remarkably efficient fusion crystal was generated. This fusion crystal demonstrated the ability to catalyze the efficient conversion of coconut oil into biodiesel (Heater et al. 2018).

**Phosphatases**. The titer of inositol monophosphatase produced in *B. subtilis* using a system mimicking the well-known *E. coli* T7 system for producing recombinant proteins, reached 5 g L^−1^ (Ye et al. 2022). The *B. subtilis* strain SCK22 was engineered by inserting the T7 RNA polymerase gene into the chromosome. In addition, two major protease genes, as well as a spore generation- and a fermentation foam generation–related genes, were knocked out. The gene for inositol monophosphatase was subcloned into plasmid pHT7, under tight control of the T7 promoter.

## Industrially relevant enzymes produced in *Streptomyces* spp.

Among streptomycetes, *S. lividans* is by far the most frequently used heterologous host, but *S. coelicolor* A3(2), *S. griseus*, *S. rimosus*, *S. hygroscopicus*, *S. venezuelae*, and *S. avermitilis* are also employed in industrial processes. Some of the industrially relevant recombinant enzymes produced in *Streptomyces* spp. are summarized in Table 2. Streptomycetes are recognized as well suited for expression of GC-rich genes; they exhibit a high secretion capacity, and certain *Streptomyces* strains display lower levels of endogenous extracellular proteolytic activity. Still, one of their drawbacks as expression hosts lies in their mycelial lifestyle, which leads to heteromorphous and viscous cultures, making them less suitable for industrial fermentation. It was shown that the enhanced expression of gene for SsgA, a protein involved in cell-wall remodeling processes, together with the right combination of promoters and signal peptides, resulted in considerable improvement of recombinant protein production in *S. lividans* (van Wezel et al. 2006; Sevillano et al. 2016).

### Proteases/peptidases

**Serine proteases**. Pernisine, thermostable serine protease derived from the hyperthermophilic Archaeaon *Aeropyrum pernix*, possesses the ability to degrade bovine prion proteins, still challenging to decontaminate in industrial facilities, was recombinantly produced in streptomycetes (Šnajder et al. 2019). The recombinant pernisine is produced by *S. rimosus*, microorganism used over decades for industrial production of oxytetracycline. Using synthetic biology techniques, a fusion of the *srT* signal sequence for *S. rimosus* protease with the codon-optimized gene for pernisine was made, resulting with the yield of 10 mg L^−1^ of proteolytically active recombinant pernisine, the amount of enzyme comparable to those expressed in *E. coli*.

**Keratinases**. While large-scale utilization of keratinase has not been fully realized, recent advancements in the field of protein engineering show the potential to facilitate the achievement of this goal in the future (Nnolim et al. 2020; Yahaya et al. 2021)*.* Different bacteria belonging to genus *Streptomyces* are natural producers of keratinases, while production of keratinases in these bacteria using recombinant techniques is still under extensive studies (Li 2021).

It was shown that overexpression of gene for keratinase in the native host could serve as promising strategy for increased keratinolytic activity. *Streptomyces* sp. SCUT-3 demonstrates efficient feather degradation, resulting in products with high amino acid content. These products can serve as a valuable nutritional source for animals, plants, and microorganisms. In order to further increase the feather degrading activity of this isolate, the protease gene *sep39* was cloned into integrative plasmid and introduced into native host *Streptomyces* sp. SCUT-3 using conjugation. With the overexpressed gene for Sep39 enzyme, keratinolytic activity in supernatant reached 64 U mL^−1^ (Table 2), improving SCUT-3’s feather degradation efficiency four times in comparison to host, *Streptomyces* sp. SCUT-3 (Li et al. 2020). In optimized, feather-containing medium, the keratinase activity in supernatant of *Streptomyces* sp. SCUT-3-*sep39* was further increased to 102 U mL^−1^.

### Glycosyl hydrolases

**Amylases.** Amylases, either as native or recombinant proteins, stand out as widely employed enzymes in the food industry. The antibiotic resistance genes are selection markers in most plasmids used for the production of recombinant proteins. But then again, the usage of antibiotics in industrial production of enzymes should be maximally minimized, not just for reduction in production costs, but also because of the emergence of antibiotic-resistant strains and complexed legal requirements. The expression platform without antibiotic resistance genes was developed and tested for production of two hydrolytic enzymes in *S. lividans* (Sevillano et al. 2017). In this system, the toxin gene was integrated into the *S. lividans* genome, while the expression plasmid carried the antitoxin gene, which served to inactivate the toxin. Within this system, the α-amylase Amy from *S. griseus* with maximum activity of 1100 U mL^−1^ and the xylanase Xys1 from *S. halstedii* JM8 reaching activity of 170 U mL^−1^ were produced (Table 2). Their production remained stable over time, confirming the effectiveness of this novel platform.

**Xylanases.** Xylanases, glycosyl hydrolases produced by streptomycetes, responsible for degradation of xylan, the most dominant component of hemicelluloses, have found application in paper and pulp industries, as well as in food production and animal feed preparation (Prakash et al. 2013). Gene for xylanase X22 from *Aspergillus nidulans*, enzyme effectively applied to enhance aroma in microvinification processes, has been cloned and expressed in heterologous host *S. lividans* (Díaz et al. 2004). The activity of xylanase reached 19 U mL^−1^ when its native signal peptide was replaced by the one for xylanase from *S. halstedii* (Table 2). Also, the oenological properties of X22 enzyme produced by *S. lividans* were comparable to the native one, suggesting the possibility to use streptomycetes recombinant system for the production of X22 at the industrial scale.

**Cellulases.** Because of the harsh conditions in many industrial applications, there is a continuous demand for more thermotolerant cellulases to be exploited for example in the food and sugar industry, where high-temperature processes such as pasteurization are employed or in the paper, waste treatment, and agricultural industries for processing cellulose-derived materials. The gene encoding a thermostable cellulase CelA from *Rhodothermus marinus* was effectively over-expressed in *S. lividans* (Hamed et al. 2017). Promoter and the sequence for signal peptide of the *S. venezuelae* subtilisin inhibitor gene (*vsi*), highly expressed/secreted endogenous protein, were used. The CelA titer reached 50–90 mg L^−1^ under optimized fermentation conditions while the obtained activity was 15 U mg^−1^ (Table 2). RNA-seq and ^13^C-based metabolic flux analysis performed on CelA-producing strain provided new insights for targeted strain improvement strategies, aiming to alleviate secretion stress and metabolic burden of the host. This approach may lead to the development of a more reliable and consistently performing *S. lividans* strain (Daniels et al. 2018).

**β-Glucosidases.** β-Glucosidases have diverse biotechnological applications in food, surfactant, biofuel, and agricultural industries (Singh et al. 2016). Gene for β-Glucosidase from *Thermobifida fusca* YX (Tfu0937) was recombinantly expressed in *S. lividans*, and the maximum enzyme activity of 4200 U L^−1^ reached (Noda et al. 2010). The expression system was based on *E. coli*-*Streptomyces* shuttle vector, with promoter, signal sequence, and terminator region from *pld* gene, coding for *Streptoverticillium cinnamoneum* phospholipase D.

**Chitinases.** Microbial chitinases possess the ability to catalyze degradation of chitin, a major component found in the shells of crustaceans, exoskeletons of insects, and fungal cell walls. Chitinase C from soil bacterium *Streptomyces coelicolor* A3(2) was expressed in *S. lividans* host (Nguyen-Thi and Doucet 2016). The final ScChiC titer of 1070 mg L^−1^ was 486-fold higher than the previously reported for the protein produced in *E. coli*, while the activity of 9215 U mg^−1^ was reached (Table 2). The ScChiC expressed in *S. lividans* could be applied as a biocatalyst, offering an appealing alternative to chemical hydrolysis of chitin. This biocatalytic approach circumvents several industrial and environmental challenges associated with chemical methods, such as acidic waste generation, low production yields, high energy consumption, and difficulties in product separation. When metagenome-sourced chitinase 53D1 gene was expressed in *E. coli*, > 80% of the recombinant protein was accumulated as inactive. To overcome this issue, three Gram-positive actinomycetes belonging to the genus *Streptomyces* were tested as hosts for 53D1 production (Berini et al. 2019). 53D1 coding gene was cloned into the multicopy *Streptomyces* plasmid under the control of the *ermE* promoter. Maximum titer of 45 mg L^−1^ was obtained with *S. coelicolor*, the unconventional but more environmentally acceptable host, suggesting the potential usage as biocontrol agent, as insecticide protein.

**Chitosanases.** There are many chitosanases among microbial enzymes, catalyzing production of different chitooligosaccharides, potentially valuable nutraceuticals, from chitosan, a partly N-deacetylated form of chitin (Lacombe-Harvey et al. 2018). The efficient production of CsnN106 chitosanase, coded by *csnN106* gene from the *Kitasatospora* sp. N106, was obtained in *S. lividans* Δ*csnR* strain (Dubeau et al. 2011). Using host with in-frame deletion of *csnR* gene and a mutated *csnN106* gene with a modified transcription operator, maximum activity of 24 U mL^−1^ was reached (Table 2), indicating that significant levels of chitosanase could be produced in inexpensive fermentation medium.

### Lipases/Esterases

**Lipases.** Lipases from *Streptomyces* spp. are some of the most important biocatalysts used for biotechnological application, for example for biodiesel production from waste or for synthesis of triacylglycerols with n-3 polyunsaturated fatty acids (Wang et al. 2017; Spasic et al. 2018). The gene for LipIAF5.2, an alkaline and thermostable lipase originated from metagenomic DNA extracted from the biomass obtained in a gelatin-enriched fed-batch reactor, was recombinantly expressed in *S. lividans* using promoter from *S. coelicolor groEL2* heat-shock gene (Meilleur et al. 2009). The high-level production of active enzyme resulted with 11.3 mg of pure active lipase per liter, the amount that enabled simple two-step purification of the lipase from the supernatant.

**Phospholipases.** Phospholipases A-D, categorized based on their substrate cleavage site and designed to modify phospholipids, essential components of biological membranes in all living organisms, are widely distributed in nature and serve diverse functions. They participate in lipid metabolism and cellular signaling in eukaryotes, while in microbes, they are involved in virulence and nutrient acquisition. Demand for industrial usage of phospholipases is constantly increasing over the last 30 years. Phospholipases find widespread application in the food industry, particularly in baking, egg yolk, and dairy industries. They are utilized to enhance the emulsifying properties by acting on phospholipids already present in the food ingredients. DENAZYME PLA2, phospholipase A2 from *S. violaceoruber*, produced via fermentation and distributed on the market by Nagase & Co., Ltd. (http://nagaseamerica.com/product/phospholipase-a2) is one example.

There are many examples of phospholipase genes recombinantly expressed in *Streptomyces* spp. The first success in producing a large amount of active and highly pure phospholipase D was obtained when *pld* gene from *Stv. cinnamoneum* was cloned into pUC702, *E. coli*, and *S. lividans* shuttle vector, expressed from its native promoter and recombinant protein secreted into the medium using its native signal sequence (Ogino et al. 2004). An expression system for the *pld103* gene from *Streptomyces racemochromogenes* sp. 10–3, in *S. lividans* as the host, was constructed (Nakazawa et al. 2011). A 2690-bp genomic DNA fragment of *S. racemochromogenes* sp. 10–3, which includes the *pld103* gene sequence along with its putative promoter and terminator region, was cloned bidirectionally into the *E. coli* and *S. lividans* shuttle vector pES. In both variants, the activity of PLD reached approximately 30 U mL^−1^, 90-fold higher activity comparing to the original PLD103 strain (Table 2). The activity of PLD from *S. halstedii*, recombinantly expressed in three different hosts, was analyzed (Tao et al. 2019). The obtained enzymatic activity when *pld* gene was expressed from thiostrepton inducible *tipA* promoter, in *S. lividans* as host, reached 68 U mL^−1^, while in *E. coli* and *Pichia pastoris*, *pld* expression was limited and exhibited noticeable toxicity to cells. To go even further, a new thiostrepton-free system for the expression of *pld* from *S. antibioticus* was proposed (Wang et al. 2022). Downstream of the engineered constitutive promoter kasO* optimized *pld* gene (G215S mutation) from *S. antibioticus*, fused to a signal peptide sequence of Sigcin from *Stv. cinnamoneum pld*, was cloned. When this construct was expressed from the integrative vector pSET152, the extracellular PLD activity reached approximately 9.85 U mL^−1^, still low from the perspective of industrial production. The alternative was to use autonomously replicating plasmids with pIJ101-based *Streptomyces* replicons. The best variant was pOJ260-*rep*3-pld* with stability gene (*sta*) inserted, and the activity obtained using strain SK-3 was 62 U mL^−1^ of PLD (Table 2), which was further improved to 87 U mL^−1^ at 32 °C in the optimized medium, giving the highest activity achieved for the recombinant PLD. Also, industrial applicability of *Streptomyces* phospholipases is highlighted in the patent application describing the synthesis of recombinant *S. antibioticus* and *Streptomyces chromofuscus* phospholipase D in *S. lividans* (Zhu et al. 2021).

### Phosphatases

** Alkaline phosphatases.** It was shown that streptomycetes could be efficient and industry-friendly hosts for the production of thermophilic proteins. Although the thermostable enzymes (thermozymes) are useful tools in biotechnology, up scaling their production in a natural host is often challenging. The alkaline phosphatase from *Thermus thermophilus* was recombinantly produced by *S. lividans* (Diaz et al. 2008). The *phoA* gene encoding the periplasmic hyperalkaline phosphatase was cloned under the control of the *Streptomyces halstedii xysA* promoter, and the resulting plasmid was introduced into *S. lividans* giving the protein activity comparable to that in *E. coli*. Moreover, it has been demonstrated that the *Streptomyces* secretion machinery efficiently recognizes the PhoA signal peptide. As a result, the secreted product is essentially pure and may not necessitate further purification for most industrial applications, making it highly desirable from an industrial standpoint.

### Transferases

** Phytases.** Bacterial phytases have a huge potential for usage in the animal feed industry due to their thermostability, higher substrate specificity, increased resistance to proteolysis, and superior catalytic efficiency compared to commercially produced fungal phytases. The variety of diverse genetic tools, including inducible and strong constitutive *Streptomyces* promoters, leader peptides known to drive efficient secretion of various *Streptomyces* genes, as well as integrative and replicative *Streptomyces* expression plasmids, were applied to produce *E. coli* AppA phytase in *S. rimosus*, the industrial producer of antibiotic oxytetracycline (Carrillo Rincón et al. 2018).

**Transglutaminases.** Transglutaminases find extensive applications in the industry, particularly in food processing, to enhance the properties of diverse proteins such as meat, soy, myosin, globulin, casein, peanut, and whey proteins. Additionally, these enzymes are also applied for protein engineering. Many attempts were made to develop expression system for transglutaminase, but in *E. coli*, transglutaminase ends in inclusion bodies, demanding a refolding process that inevitably reduces the enzymatic activity, while in other expression systems, transglutaminase requires pre- and/or pro-sequences. Gene for *S. hygroscopicus* transglutaminase (TGase) was successfully expressed in *S. lividans*, using its endogenous promoter, signal peptide, and terminator (Liu et al. 2016). The pro-TGase was correctly processed into active TGase reaching activity of 1.8 U mL^−1^. Recombinant production of TGase was further improved when the negative element of the promoter was deleted and gene codons for transglutaminase optimized so the activity of TGase reached maximum of 5.73 U mL^−1^.

## Final considerations and future prospects

An increasing array of food, detergent, and pharmaceutical compounds is being manufactured using microorganisms or their enzymes. Since the demand for sustainable and eco-friendly products continues to rise, microorganisms are recognized as valuable green factories capable for producing food and cosmetic products among others. Nevertheless, the scale-up and commercialization of microbially derived compounds is still constrained by factors like the suboptimal economic aspects, performances of microbial strains and/or biotechnological processes. The simplicity of cultivation, the availability of a wide range of well-established genetic manipulation tools, and its inherent ability to efficiently accommodate and express foreign proteins, still make *E. coli* favorable as expression host. However, there is a growing demand to shift from processes reliant on enzymes and industrial microbial hosts to evolved microbial hosts and enzymes. This transition aims to enhance the robustness and flexibility of bio-based production processes, leading to improved outcomes. Numerous molecular biology techniques and strategies have been designed to effectively optimize microbial strains and processes. The ideal minimized chassis for recombinant protein production is anticipated to exhibit robust growth, sufficient cellular energy, well-defined metabolic profiles, and genetic stability. In recent years, CRISPR-Cas9-based molecular tools, offering precision and flexibility in handling, have been increasingly adapted for various applications in both *Bacillus* and *Streptomyces* spp., and it is to expect that these tools will be successfully used for future host design to obtain maximal production of enzymes that could fulfill industrial needs, considering also the production costs. Indeed, both *Bacillus* and *Streptomyces* spp. demonstrate valuable characteristics as non-conventional hosts for recombinant protein production, manifested in cost-effectiveness, high yields of secreted protein products, and their safety of use. Due to their significant large-scale fermentation capacity and proficiency in bulk manufacturing of enzymes, *Bacillus* strains continue to be the preferred and robust production platform for heterologous proteins. Nevertheless, decades of practice with *Streptomyces* spp. used as powerful antibiotic producing strains and growing utilization of specific biocatalysts expressed in *Streptomyces* spp. make them a promising alternative. Indeed, *Bacillus* and *Streptomyces* spp. are robust hosts for enzyme production and using them offers additional degree of flexibility of both products and processes.

## Supplementary Information

Below is the link to the electronic supplementary material.

Supplementary file1 (PDF 133 KB)

## Data Availability

This publication incorporates all data that were either generated or analyzed during the study, including those presented in the supplementary material.
