# Supplementary material for: Bacillus and Streptomyces spp. as hosts for production of industrially relevant enzymes
Source: Appl Microbiol Biotechnol. 2024 Jan 30;108:185. doi: 10.1007/s00253-023-12900-x (PMC10827964; doi:10.1007/s00253-023-12900-x)
Supplement: Supplementary file 1 [file 253_2023_12900_MOESM1_ESM.pdf]

Supplementary Material

Bacillus and _Streptomyces_ spp. as hosts for production of industrially relevant enzymes

Sandra Vojnovic[a*], Ivana Aleksic[a], Tatjana Ilic-Tomic[a], Milena Stevanovic[a], Jasmina Nikodinovic-Runic[a*]

[a]*Institute of Molecular Genetics and Genetic Engineering, University of Belgrade, Vojvode Stepe 444a, 11042 Belgrade 152, Serbia*

[*]Corresponding authors. Tel.: +381 11 397 6034; fax: +381 11 397 5808 (J. Nikodinovic-Runic, S. Vojnovic)

E-mail addreesss: jasmina.nikodinovic@imgge.bg.ac.rs (J. Nikodinovic-Runic); sandravojnovic@imgge.bg.ac.rs (S. Vojnovic)

ORCID No:

Sandra Vojnovic 0000-0002-5083-4287

Ivana Aleksic 0000-0001-5635-0024

Tatjana Ilic-Tomic 0000-0001-7198-2855

Milena Stevanovic 0000-0002-5590-685X

Jasmina Nikodinovic-Runic 0000-0002-2553-977X

Table S1. Representative industrially relevant enzymes produced by microorganisms and commercially available at https://www.sigmaaldrich.com/

**Protein class/Application**	**Protein**	**Source**	**Heterologous host**	**Activity**	**Cat. No.**
**Proteases/peptidases EC 3.4.X.Y**<br><br>Pharmaceutical, Leather, Food and Waste processing industry; Animal feed preparation; Life sciences (Proteomics; Protein structure analysis and sequence analysis)	Alkaline protease	*Aspergillus saitoi*	-	≥0.6 U mg^{-1}	P2143
	Flavourzyme	*Aspergillus oryzae*	-	≥500 U g^{-1}	P6110
	Proteinase	*Aspergillus melleus*	-	≥3 U mg^{-1}	P4032
	Carboxypeptidase Y	*Saccharomyces cerevisiae*	-	≥50 U mg^{-1}	C3888
	Rennin	*Mucor miehei*	-	~0.1 U mg^{-1}	83553
	Pronase E	*Streptomyces* sp.<br>*Streptomyces griseus*	-	≥15 U mg^{-1}<br>≥3.5 U mg^{-1}	P0652<br>P5147
	Aminopeptidase I	*S. griseus*	-	≥200 U mg^{-1}	A9934
	Promatex	*Bacillus* sp.	-	≥1.5 U mg^{-1}	P0029
	Subtilisin A	*Bacillus licheniformis*	-	-	P3910
	Neutrase 0.8L	*Bacillus amyloliquefaciens*	-	≥800 U mg^{-1}	P1236
	Keratinase	*B. licheniformis*	*E. coli*	300-1000 U mg^{-1}	K4519
	Alkaline Protease	*B. licheniformis*	-	7.0-14.0 U mg^{-1}	P8038
	Thimet Oligopeptidase	*B. licheniformis*	*E. coli*	-	T7705
	Acidic protease	*Rhizopus* sp.	-	≥0.2 U mg^{-1}	P0107
	Alpha-lytic protease	*Lysobacter enzymogenes*	-	≥0.0005 U mg^{-1}	A6362
	Endoproteinase Lys-C	*L. enzymogenes*	-	≥200 U mg^{-1}	324715
	Endoproteinase Glu-C	*Staphylococcus aureus*	-	≥15 U mg^{-1}	324713
	Endoproteinase Glu-C	*Staphylococcus aureus* V8		500-1 000 U mg^{-1}	P2922
	Thermolysin	*Geobacillus stearothermophilus*	-	30-350 U mg^{-1}	P1512
	Aminopeptidase	*Vibrio proteolyticus*	*E. coli*	50-100 U mg^{-1}	A4987
	Proline Specific Endopeptidase	*Flavobacterium* sp.	-	≥5 U mg^{-1}	E1411
	Endopeptidase Lys-C	*Achromobacter lyticus*	-	≥2 U mg^{-1}	324796
	Endoproteinase Asp-N	*Pseudomonas fragi*	-	≥20 U g^{-1}	324708
	Carboxypeptidase G	*Pseudomonas* sp.	-	≥3 U mg^{-1}	C9658
	Methionine Aminopeptidase	*Pyrococcus furiosus*	*E. coli*	0.5 U mg^{-1}	M6435
	Pyroglutamate Aminopeptidase	*P. furiosus*	*E. coli*	≥5 U mg^{-1}	P6236
	Clostripain	*Clostridium histolyticum*	-	≥20 U mg^{-1}	C0888
	Aminopeptidase	*Aeromonas proteolytica*	-	50-150 U mg^{-1}	A8200
**Glycosyl hydrolases EC 3.2.1.X**<br><br>Food industry, Agricultural industry (improvement the nutritive value of poultry feed, control of	Cellulase	*Aspergillus niger*	-	~0.8 U mg^{-1}	22178
	Lyticase	*Arthrobacter luteus*	-	≥2 U g^{-1}	L2524
	Chitinase	*S. griseus*<br>*Trichoderma viride*	-	>200 U mg^{-1}<br>≥600 U g^{-1}	SAE0158<br>C8241

pathogens); Pharmaceutical industry (preparation of chitooligosaccharides and N-acetyl D glucosamine); Treatment of chitinous waste; Life sciences (Isolation of protoplasts from fungi and yeast)					
**Lipase/Esterase**<br>**EC 3.1.1.X**<br><br>The synthesis of biosurfactants, organic chemical processing, application in dairy industry, oleochemical industry, detergent formulations, paper, cosmetics, nutrition, and the pharmaceutical industry; The synthesis of dextran fatty acid esters for various industrial applications; Producing biodiesel from waste; Biocatalysis; Life sciences; Diagnostic application	Lipase	*A. niger*<br>*Rhizomucor miehei*<br>*Candida antarctica*<br>*Candida rugosa*<br>*Chromobacterium viscosum*<br>*Rhizopus oryzae*<br>*Rhizopus niveus*<br>*Pseudomonas cepacia*<br>*M. miehei*<br>*Mucor javanicus*	-	200 U mg^{-1}<br>≥20000 U g^{-1}<br>~0.3 U mg^{-1}<br>≥700 U mg^{-1}<br>≥2.5 U g^{-1}<br>≥30 U mg^{-1}<br>≥1.5 U mg^{-1}<br>≥30 U mg^{-1}<br>≥4 U g^{-1}<br>≥300 U mg^{-1}	62301<br>L4277<br>02569<br>L1754<br>437707<br>80612<br>62310<br>62309<br>L9031<br>L8906
	Lipoprotein Lipase	*Burkholderia sp.*	-	≥50 U g^{-1}	L9656
	Amano Lipase	*Pseudomonas fluorescens*	-	≥20 U g^{-1}	534730
	Esterase	*Bacillus stearothermophilus*<br>*Bacillus subtilis*<br>*Pseudomonas fluorescens*	*E. coli*	≥4.0 U mg^{-1}<br>≥10 U mg^{-1}<br>≥4 U mg^{-1}	79302<br>96667<br>75742
	Esterase	*R. oryzae*	-	> 20 U g^{-1}	79208
	Cholesterol Esterase	*P. fluorescens*	-	≥10 U g^{-1}	C9281
**Oxidoreductase**<br>**EC 1.X.Y.Z**<br><br>Biofuel production, teeth whitening, textile dyeing, and in other applications that require the removal of oxygen; Waste and water treatment; Delignification, paper processing, waste detoxification, Food and pharmaceutical industries; glucose biosensors	Laccase	*Agaricus bisporus*<br>*Trametes versicolor*<br>*Aspergillus sp.*	-	≥4 U/mg<br>≥0.5 U/mg<br>-	40452<br>38429<br>SAE0050
	Manganese peroxidase	*Phanerochaete chrysosporium*	-	≥10000 U mg^{-1}	93014
	Glucose oxidase	*A. niger*	-	100000 -250000 U g^{-1}	G7141
	ω-transaminase	*Aspergillus fumigatus*<br>*Aspergillus terreus*<br>*Neosartorya fischeri*	*E. coli*	≥0.20 U mg^{-1}<br>≥0.10 U mg^{-1}<br>≥0.4 U mg^{-1}	77087<br>40972<br>93006
**Phosphatases**<br>**EC 3.1.3.X**<br><br>Different applications in life sciences (ELISA, Western blotting, and histochemical detection)	Phosphatase, Alkaline	*E. coli*	-	30-60 U mg^{-1}	P5931
	Streptavidin−Alkaline Phosphatase	*Streptomyces avidinii- E. coli*	*E. coli*	≥700 U mg^{-1}	S2890
	Protein A, Alkaline Phosphatase Conjugate	*S. aureus, E. coli*	*E. coli*	-	539251
**Nucleases**<br>**EC 3.1.3X.Y**<br><br>Different applications in life sciences (protein extraction, microbiome	Nuclease S7	*S. aureus*	-	~15000 U mg^{-1}	10107921001
	Nuclease P1	*Penicillium citrinum*	-	≥200 U mg^{-1}	N8630
	Nuclease	*S. aureus*	-	≥15000 U mg^{-1}	492899
	Nuclease micrococcal	*S. aureus*	-	100-<br>300 U mg^{-1}	N3755

research, and bioprocessing, degradation of DNA and RNA, RNA sequencing experiments, nucleic acids structural analysis,); industrial production of 5′-mononucleotides	Benzonase® Nuclease	*Serratia marcescens*	*E. coli*	≥250 000 U mL^{-1}	E1014
	Nuclease S1	*A. oryzae*	-	≥100000 U mL^{-1}	N5661
	Turbonuclease	*S. marcescens*	*E. coli*	≥200000 U mL^{-1}	T4330
**Polymerases EC 2.7.7.7**<br><br>Life sciences: Polymerase chain reaction	Taq DNA Polymerase	*Thermus aquaticus*	*E. coli*	5000 U mL^{-1}	D4545
	KOD DNA Polymerase	*Thermococcus kodakarensis*	*E. coli*	2500 U mL^{-1}	71085
